# Evidence on the Relationship Between Emotional Intelligence and Risk Behavior: A Systematic and Meta-Analytic Review

**DOI:** 10.3389/fpsyg.2022.810012

**Published:** 2022-02-09

**Authors:** María T. Sánchez-López, Pablo Fernández-Berrocal, Raquel Gómez-Leal, Alberto Megías-Robles

**Affiliations:** Department of Basic Psychology, Faculty of Psychology, University of Málaga, Málaga, Spain

**Keywords:** emotional intelligence, risk behavior, risk domain, systematic review, meta-analysis

## Abstract

The aim of the present study was to carry out a qualitative and quantitative synthesis of the existing literature studying the relationship between emotional intelligence and risk behavior. We conducted a systematic review and meta-analysis of the scientific evidence available relating both constructs. Particular attention was paid to identifying possible differences in this relationship as a function of the different conceptualizations of EI and the risk domain. The study was conducted following the Cochrane and PRISMA guidelines. Our results revealed a significant negative relationship between EI and health-related risk behaviors. However, this relationship was not observed in other risk domains such as finance and gambling. The relationship between EI and risk behavior differed according to the risk domain studied, which supports the notion that risk is a domain-specific construct. The results associated with the health-related risk behaviors are consistent with existing literature about the positive impact of emotional abilities on the health domain. A more complete understanding of the emotional mechanisms that underlie risk behavior could help to establish action guidelines and improve programmes to prevent and reduce the negative effects of risk behavior on our society.

## Introduction

Emotions are fundamental in our lives, as they form part of the basis of our behavior and help us to make decisions, guiding our attention, memory, motivation, and learning (Dolan, 2002; Pessoa, 2008). In this regard, Emotional Intelligence (EI) combines two concepts that for years seemed to represent an oxymoron—cognition and emotion. EI refers to the ability to identify, understand, use and regulate one's own emotional states and those of others (Mayer et al., 2016). Higher EI abilities have been positively related to various aspects of life such as physical and psychological health (Martins et al., 2010; Domínguez-García and Fernández-Berrocal, 2018; Megías et al., 2018c), optimal coping abilities (Salovey et al., 1999, 2002), appropriate social interactions (Lopes et al., 2011), lower levels of aggressive behavior (Megías et al., 2018b; Gómez-Leal et al., 2020) or greater wellbeing and vital satisfaction (Johnson et al., 2009; Andrei and Petrides, 2013; Laborde et al., 2014). Emotion also plays a central role in risk behavior (Loewenstein et al., 2001; Reyna, 2004; Slovic, 2010). It is well-known that people adapt their behavior in risk situations not only through a rational process but also by following their emotions (Slovic et al., 2004; De Martino et al., 2006; Rivers et al., 2008). However, whilst there is an extensive body of literature on the influence of emotion on risk behavior, the relationship between EI and risk behavior has received relatively little attention.

Risk behavior is defined as any behavior that generates a probability of objective or subjective loss, this loss being significant for the individual (Yates and Stone, 1992). Engaging in this kind of behavior often poses a threat to fundamental needs such as our health, safety, or wellbeing (Pellmar et al., 2002; WHO, 2009, 2018). Some examples include unsafe sexual activities, substance abuse, risky driving, and gambling with large amounts of money. All theoretical models of risk behavior include emotion as a fundamental factor in these behavioral choices (Damasio, 1994; Loewenstein et al., 2001; Reyna, 2004; Slovic et al., 2004). For example, Slovic et al. (2004, 2007) present risk as a feeling rather than as a statistical representation, and they coined the term *affect heuristic* to explain how stimulus-affect associations determine our behavior in many risk situations. In addition, another important factor to take into account is that the contexts where risk situations take place are usually characterized by a strong emotional charge, which influences our behavior (Ditto et al., 2006; Gutnik et al., 2006; Rivers et al., 2008; Megías et al., 2011). An emotional state of positive valence and high arousal—whether this is present prior to the contextual situation or generated by the situation itself—has been shown to encourage both unsafe sexual intercourse and increased gambling behavior (Sánchez et al., 2001; Ariely and Loewenstein, 2006; Cyders and Smith, 2008; Haase and Silbereisen, 2011). Evidence of the integration between emotional and cognitive processes in risk behavior has also been revealed at a neural level (Vorhold, 2008; Mohr et al., 2010; Megías et al., 2015). Research has shown that neural representations of risk activate brain areas involved in emotional processing such as the anterior insula, the amygdala, and the ventromedial prefrontal cortex, among others (Vorhold, 2008; Mohr et al., 2010; Megías et al., 2015, 2018a).

Given the key role that emotion plays in risk behavior, it is expected that our ability to perceive, use, understand, and manage our emotions influence our tendency to engage in risk-taking. These abilities should act as a protective factor for risk behavior, that is, individuals with better abilities should show a tendency to engage in fewer risk behaviors. As already described, the concept of EI encompasses all these emotional abilities (Mayer et al., 2016). Some research studies (albeit scarce) have aimed to explore the relationship between EI and risk behavior (Rivers et al., 2013; Fernández-Abascal and Martín-Díaz, 2015; Lando-King et al., 2015; Hayley et al., 2017); however, the literature does not present conclusive results and no systematic review has yet been conducted to synthesize the results of these investigations.

One challenge inherent to the study of risk behavior is that risk is a domain-specific construct (Weber et al., 2002). Risk-taking does not constitute a rigid pattern of behavior—rather, it is expressed in different ways across various areas of our lives (e.g., social, finance, health, security, or recreational). An individual can have a risky attitude in some areas and not in others. For instance, one might engage in unsafe sex and drunk driving but be conservative when dealing with financial investments. Thus, when studying attitudes toward risk, we should always take into account the context in which the decision is made. Accordingly, previous research has revealed how, depending on the contextual situation, different personality traits influence the tendency to take risks (Blais and Weber, 2006; Lozano et al., 2017). For example, impulsivity-related traits such as high levels of positive urgency predict increases in risky sexual practices and risky driving behaviors (Zapolski et al., 2009; Baltruschat et al., 2020), whilst high levels of negative urgency appear to be more strongly associated with problematic alcohol use, self-harming behaviors, or eating disorders (Dir et al., 2013; Mallorquí-Bagué et al., 2020). Likewise, the sensation seeking trait has been related to recreational risks rather than financial risks (Lozano et al., 2017). As is the case with these personality traits, the protective role of EI in risk-taking behavior could depend on the risk domain being studied.

It is also important to note that the concept of EI in the literature has been investigated from three different approaches, depending on the construct–method pairing: self-report mixed model, self-report ability model, and performance-based ability model (Joseph and Newman, 2010). The self-report mixed model understands EI as a broad construct composed of various measures of personality and affect, which are assessed using subjective self-report measures. The self-report ability model considers EI as a form of mental ability based on emotional aptitudes and employs subjective self-report measures through which people assess the perception of their own EI abilities. Finally, the performance-based ability model also treats EI as a form of mental ability but assessed EI in a more objective manner through instruments where individuals must solve questions with correct and incorrect responses. Although the three models are popular in the EI literature, research has shown that the performance-based ability model is less sensitive to subjective and social desirability bias (Brackett et al., 2006; Webb et al., 2013) and is more consistent in predicting general behavior (Mayer et al., 2016; Gutiérrez-Cobo et al., 2017). These differences in the definition of the construct and assessment method could result in discrepant findings in the study of the relationship between EI and risk behavior.

The purpose of the present study was to conduct a systematic review and meta-analysis that allows for a qualitative and quantitative synthesis of the scientific evidence available on the relationship between EI and risk behavior. Although it is well-known that EI promotes numerous benefits in a wide variety of psychological and behavioral variables, to date, research studying the role of EI as a protective factor against risk-taking behavior is limited, and there is no systematic review that summarizes the existing literature and provides a complete overview of this phenomenon. We propose the existence of a negative relationship between EI and risk behavior, however, given some of the mixed findings reported in the literature, we pay particular attention to determining whether these differences among studies arise as a function of the risk domain where the behavior is performed and the conceptual model of EI employed. A more in-depth understanding of this relationship could help to improve actions aimed at preventing and reducing the effects of risk behavior on our society.

## Methods

The systematic review and meta-analysis were conducted according to Cochrane guidelines (Higgins and Green, 2011).

### Search Methods for Identification of Studies

#### Information Sources and Search Terms

In order to identify all eligible studies that associate EI with risk behavior, a comprehensive systematic literature search was conducted using the PsycINFO, PubMed and Scopus databases. The literature search was performed during April 2020. The searches included articles published between 1990 (inception of the concept of EI) and April 2020 containing in the title, abstract or keywords the term “emotional intelligence” together with one of the following terms: “risky behavior,” “risk behavior,” “risky behaviour,” “risk behaviour,” “risk taking,” and “risk perception.” The search was restricted to only these terms in order to ensure that the selected articles assessed the constructs of EI and risk behavior by instruments designed specifically for this aim. In addition, hand searches were conducted on the reference lists of the selected articles to check that no studies were overlooked (no new articles were obtained from reference lists).

#### Eligibility Criteria

The aim of the search strategy was to locate and select for inclusion all those studies investigating the relationship between EI and risk behavior that have been published in peer-reviewed scientific journals before April 2020. For inclusion, the studies were required to assess EI through instruments based on one of the three theoretical models of EI (Joseph and Newman, 2010), and work with instruments specifically designed to assess risk behavior, understanding it as a decision-making process in which the individuals face the likelihood of incurring an objective or subjective loss, which must be of significance to said individuals (Yates and Stone, 1992). The exclusion criteria were: (a) studies not published in scientific journals such as theses, books, or reports; (b) theoretical, qualitative, or review articles; (c) articles written in a language other than English or Spanish; (d) studies that did not examine behavior that meets the definition of risk behavior; (e) studies assessing EI through instruments that are not considered measures of EI; (f) studies that used an EI questionnaire, not to evaluate EI, but a single aspect or ability associated with EI, for example emotion regulation; (g) studies that examined EI and risk behavior, but not the relationship between them.

### Data Collection and Analysis

#### Selection of Studies

**Figure 1:**
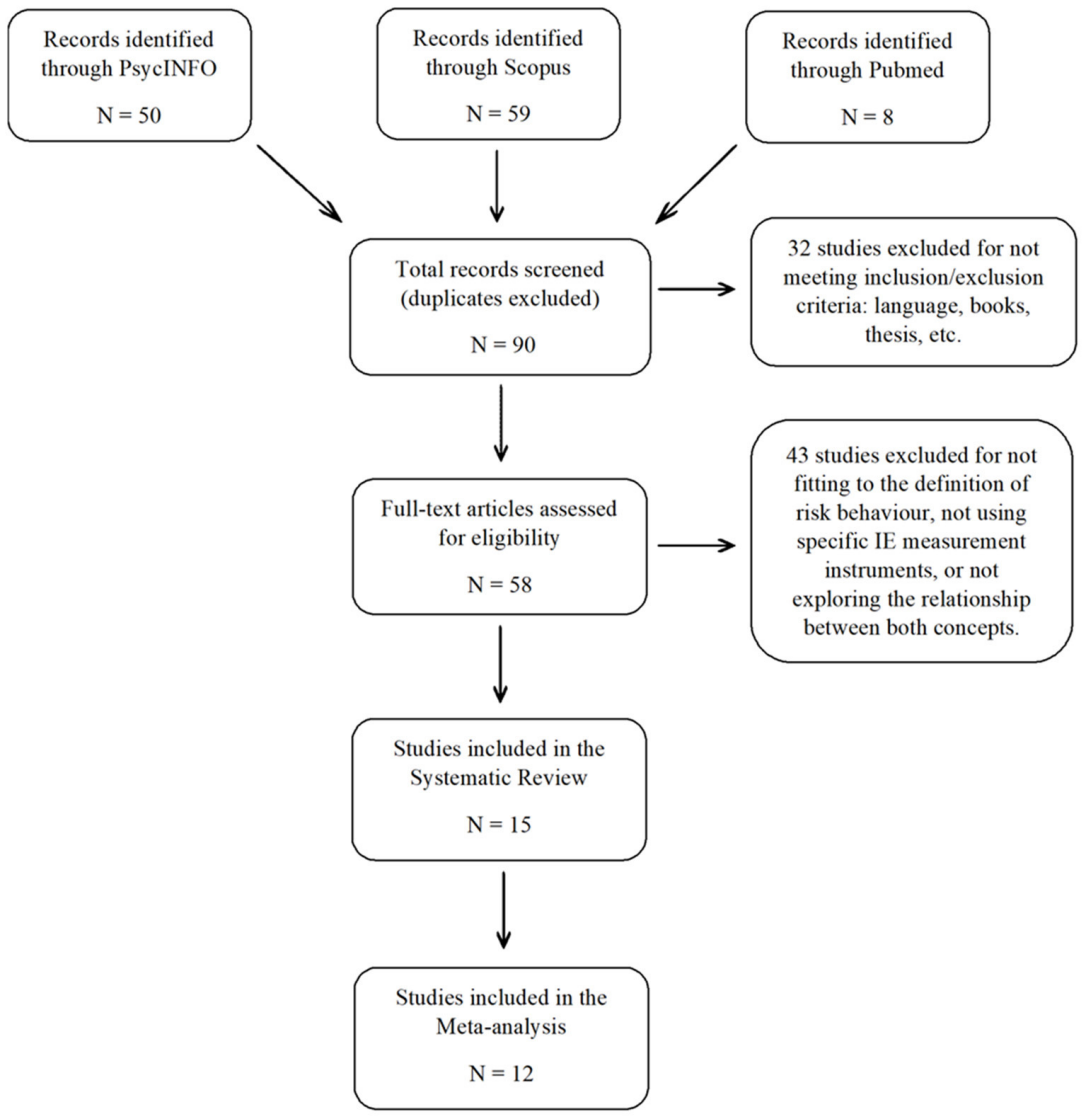
Flow chart of the search process.

Two review authors (M.T.S.L. and A.M.R.) working independently, carried out the search and examined the selected studies according to inclusion and exclusion criteria. Discrepancies were resolved through discussion with other two authors (P.F.B. and R.G.L.) The results of the literature search and study selection are shown (following PRISMA guidelines) in the flow chart presented in Figure 1 (Moher et al., 2009).

A total of 117 articles were identified by entering the search terms in the databases. After removing duplicates, 90 articles were obtained to screen by abstract. Of these, 58 articles were selected for a full-text review based on the exclusion criteria. Finally, 15 studies relating EI to risk behavior and meeting the inclusion and exclusion criteria were included in the systematic review.

Of the 90 total articles examined, 75 were removed based on the following exclusion criteria: 17 articles not published in scientific journals, 8 theoretical or review articles, 7 articles written in a language other than English or Spanish, 4 articles understanding risk behavior as a behavior external to the individual and not as a decision-making process that culminates in risk behavior (e.g., perceived risk of a terrorist attack or risk of revictimization), 14 articles that did not use a specific EI measurement instrument, 18 articles that investigated certain aspects related to emotional abilities but not EI *per se* (e.g., facial recognition of emotional expressions or emotional regulation strategies), and 7 articles that evaluated EI and risk behavior but did not explore the link between the two concepts.

#### Data Extraction

For each of the selected articles, we extracted a set of data related to authors, year of publication, sample size, mean age, gender, country of origin of the study, risk behavior and EI measurement instruments, risk behavior domain, EI model, primary outcomes, and effect size (see Table 1). Pearson's *r* correlation coefficient was used to determine effect size. When articles presented more than one measurement instrument for EI or risk behavior, the results for these instruments were described individually.

**Table 1:** Main characteristics of the studies included in the systematic review.

**References**	**Sample size**	**Mean age (years)**	**Percentage of men**	**Study country**	**Risk behavior instrument**	**Risk domain**	**EI instrument**	**EI model**	**Primary outcomes**	**Effect size [Pearson's correlation coefficient (r)]**
Alipour and Mijani (2013)	285	No reported	No reported	Iran	Researcher-built questionnaire	Finances	Shrink questionnaire	Mixed	Significant positive relationship between EI and risk behavior.	0.15
Anwar et al. (2016)	225	17.41	48.00%	Pakistan	HRBQ	Health	SEI	Mixed	Significant negative relationship between EI and risk behavior.	−0.48
Dinç Aydemir and Aren (2017)	496	No reported (20 years or above)	55.40%	Turkey	Researcher-built questionnaire	Finances	SSRI	Self-report ability	No relationship between EI and risk behavior. Significant positive relationship between EI and risk behavior when this relationship was included in a structural equation model along with the variables of locus of control, risk aversion, and financial literacy.	0.00
Fernández-Abascal and Martín-Díaz (2015)	855	34.27	21.98%	Spain	HBC	Health	TMMS and TEIQue	Self-report ability and Mixed	Significant negative relationship between the TMMS dimension of clarity and risk behavior. Significant positive relationship between the TMMS dimension of attention and risk behavior. Significant negative relationship between the TEIQue dimensions of emotionality and self-control and risk behavior. The remaining dimensions of the TMMS and TEIQue were no related risk behavior.	For TMMS EI instrument: −0.02 For TEIQue EI instrument: −0.09
Hayley et al. (2017)	179	29.85	55.00%	Australia	BDDS and DDDI	Health	SUEIT	Self-report ability	No relationship between EI and risk behavior.	0.01
Lana et al. (2015)	275	22.40	11.60%	Spain	Researcher-built questionnaire	Health	SSRI	Self-report ability	The group of participants scoring higher in the risk behaviors of excessive alcohol consumption and unsafe sex showed lower EI. The risk behavior of illicit drug use was not associated with EI.	Not applicable.
Lando-King et al. (2015)	253	15.60	0.00%	USA	Researcher-built questionnaire	Health	BarOn EQ-i: YV	Mixed	Significant negative relationship between the BarOn EQ-i: YV dimensions of intrapersonal and interpersonal skills and number of sex partners. Significant negative relationship between the BarOn EQ-i: YV dimension of stress management and inconsistent condom use. The remaining dimensions of the BarOn EQ-i: YV were no related to number of sex partners or inconsistent condom use.	For the risk behaviors of “number of sex partners”: 0.16 For the risk behaviors of “inconsistent condom use”: −0.05
Malinauskas et al. (2018)	1,214	22.36	49.17%	Lithuania	HBC	Health	SSRI	Self-report ability	Significant positive relationship between all the dimensions of the SSRI and the risky driving behavior. Significant negative relationship between the SSRI dimensions of optimism, appraisal, and utilization and the risk behavior of substance abuse. No relationship between the SSRI dimension of social skills and substance abuse.	For the risk behaviors of “traffic risk taking”: 0.16 For the risk behaviors of “substance risk taking”: −0.05
Micklewright et al. (2015)	34	39.9	94.12%	UK	DOSPERT	General risk perception	SSRI	Self-report ability	The group of higher risk-perceivers showed higher EI compared to the group of lower risk-perceivers.	Not applicable.
Panno (2016)	94	17.23	79.00%	Italy	Cold CCT	Gambling	TEIQue-ASF	Mixed	Significant positive relationship between EI and risk behavior.	0.25
Panno et al. (2015)	158	21.64	24.00%	Italy	Hot CCT	Gambling	TEIQue-SF	Mixed	No significant direct relationship between EI and risk behavior. Significant positive indirect relationship between EI and risk behavior *via* negative mood and anticipated fear.	0.09
Rivers et al. (2013)	243	No reported (between 18 and 19)	25.10%	USA	CSLSS	Health	MSCEIT	Performance-based ability	Significant negative relationship between EI and the risk behaviors of substance abuse, adjustment problems, and aggressive behavior.	For the risk behaviors of “substance abuse”: −0.18 For the risk behaviors of “adjustment problems”: −0.16 For the risk behaviors of “aggressive behavior”: −0.25
Vaughan et al. (2019)	269	21.80	57.62%	Ireland	CGT	Gambling	SSRI	Self-report ability	Significant negative relationship between the four dimensions of the SSRI and risk behavior.	−0.2
Yip and Côté (2013)	52	24.00	37.00%	USA	IGT	Gambling	MSCEIT	Performance-based ability	No relationship between EI and risk behavior.	Not applicable.
Zavala and López (2012)	829	13.60	47.50%	Mexico	MACI	Health	BarOn EQ-i: YV	Mixed	Significant negative relationship between EI and risk behaviors associated with eating disorder and substance abuse.	For the risk behaviors of “eating disorders”: −0.20 For the risk behaviors of “substance abuse”: −0.29

*HRBQ, Health Risk Behavior Questionnaire; HBC, Health Behavior Checklist; BDDS, Distracted Driving Scale; DDDI, Dangerous Driving Index; DOSPERT, Domain Specific Risk Taking; CCT, Columbia Card Task; CSLSS, College Student Life Space Scale; CGT, Cambridge Gambling Task; IGT, Iowa Gambling Task; MACI, Millon Adolescent Clinical Inventory; SEI, Scale of Emotional Intelligence; SSRI, Schutte Self-Report Inventory; TMMS, Trait Meta Mood Scale; TEIQue/-SF/-ASF, Trait Emotional Intelligence Questionnaire/-Short Form/ -Adolescent Short Form; SUEIT, Swinburne University Emotional Intelligence Test; BarOn EQ-i: YV, Bar-On Emotional Quotient Inventory: Youth Version; MSCEIT, Mayer-Salovey-Caruso Emotional Intelligence Test*.

*For those studies that did not show a global score of EI, primary outcomes were reported separately for each EI dimension and effect sizes were averaged across EI dimensions within each study in order to provide an approximate effect size for the global EI (see Results of the meta-analysis section)*.

#### Data Synthesis and Statistical Analysis

The articles that fulfilled the inclusion and exclusion criteria were synthesized using a qualitative narrative approach and a quantitative meta-analysis. We decided to undertake a qualitative synthesis along with the meta-analysis to better address the heterogeneity of the selected articles. Many of the studies varied in their assessment methods, characteristics of the variables, and use of covariates, while some also included additional designs to those aimed at analyzing the primary relationship of interest. Thus, although a qualitative synthesis provides less objective results than a meta-analysis, it allows us to carry out a more in-depth individual discussion of each study.

The qualitative synthesis was based on the description of the data and results collected from the systematic review. For those studies in which the measurement instruments of EI and risk behavior did not provide a global score, but assessed the construct through several dimensions, the results for each of the dimensions were considered individually (see Table 1). For the risk measurement instruments, those dimensions that did not explicitly assessed risk behavior (e.g., feelings of anxiety) were excluded.

To conduct the meta-analysis, effect sizes were extracted from those articles containing such information. As already mentioned, we used Pearson's *r* correlation coefficient as a measure of effect size. When Pearson's *r* was not available in the article, we tried to compute this coefficient from descriptive or inferential statistics. However, these articles did not include the necessary information and we contacted the corresponding authors *via* email in order to request these values (Pearson's *r*). For those studies assessing several risk behaviors (e.g., traffic risk taking and substance risk taking) or using more than one EI measuring instrument, the individual effect size of each of these outcomes was included in the meta-analysis. In order to handle dependency among effect sizes within these studies, a three-level meta-analytic model was conducted (Van Den Noortgate et al., 2013). The three-level approach includes an additional level of analysis in which within-study effect sizes are nested prior to the between-study estimation. Moreover, there were articles that did not provide a global score of EI, but individual scores of the dimensions that comprise the EI construct. In these cases, we averaged the effect sizes of the EI dimensions within each study in order to get an approximate result to the global EI. With respect to the meta-analytic model used, given the differences across studies in characteristics of the sample and methods, a random-effects approach (a three-level random effects model) was conducted to pool the effect sizes (Hedges and Vevea, 1998; Viechtbauer, 2010). The model was estimated using restricted maximum likelihood (REML), since this procedure provides a good balance between unbiasedness and efficiency, particularly for small sample sizes (Viechtbauer, 2005). Heterogeneity among studies was evaluated using Cochran's Q statistic and potential publication bias was evaluated by Egger's test Rosenthal's Fail-Safe N test (Egger et al., 1997; Viechtbauer, 2010). The statistical analyses were conducted by the metafor package implemented in R software version 3.6 (The R Foundation for Statistical Computing, Vienna, Austria; http://www.r-project.org).

#### Data Availability Statement

The raw data file included in the meta-analysis is available from the corresponding author on request. Furthermore, effect sizes for each study can be found in Table 1.

## Results

### Search Results and Characteristics of the Included Studies

Fifteen articles meeting the inclusion and exclusion criteria were included in the systematic review. Table 1 provides an overview of the main characteristics of the studies. The total number of participants across the 15 articles was *n* = 5,461 (mean percentage of men across studies = 43.18%; mean age across studies = 22.97 years). The distribution of the nationalities was: USA (three studies), Italy and Spain (two studies each), Australia, Iran, Ireland, Lithuania, Mexico, Pakistan, Turkey, and UK (one study each).

The selected articles measured EI from the three approaches proposed by Joseph and Newman (Joseph and Newman, 2010). Six articles used a EI measurement instrument based on mixed models: The Trait Emotional Intelligence Questionnaire, including its reduced version and adaptation for adolescents (TEIQue-SF and TEIQue-ASF; Petrides and Furnham, 2001; Petrides, 2009), the Bar-On Emotional Quotient Inventory: Youth Version (BarOn EQ-i: YV; Bar-On and Parker, 2000), the Shrink Emotional Intelligence Questionnaire (Yadegar Tirandaz et al., 2020) and the Scale of Emotional Intelligence (SEI; Batool and Khalid, 2009). Six articles used a EI measurement instrument based on the self-reported ability model: The Schutte Self-Report Inventory (SSRI; Schutte et al., 1998) and the Swinburne University Emotional Intelligence Test (SUEIT; Palmer and Stough, 2001). With regard to the performance-based ability model, two articles employed a EI measurement instrument based on this approach; specifically, these studies used the Mayer-Salovey-Caruso Emotional Intelligence Test (MSCEIT; Mayer et al., 2002). In addition, there was an article that employed measures of both the mixed model and self-reported ability model (TEIQue and TMMS; Salovey et al., 1995; Petrides, 2009).

The instruments employed to measure risk behavior varied considerably between studies. We found 16 different measurement instruments (see Table 1), both self-report and behavioral measures, which provided results from the following risk domains: risk behaviors associated with health (e.g., sexual risk behavior, illicit substance and alcohol abuse, and risky driving behavior) and risk behaviors associated with finance and gambling. Some articles assessed several types of risk behavior in the same study and one article assessed risk perception in general (including different domains in a single risk score).

### Qualitative Synthesis of the Systematic Review

Of the 15 articles included in the systematic review, 13 showed some statistically significant relationship between EI and risk behavior (see Table 1). Two articles did not find any significant relationship (Yip and Côté, 2013; Hayley et al., 2017). Focusing on those articles that reported significant results in exclusively one direction, we can observe that five revealed a negative relationship (Zavala and López, 2012; Rivers et al., 2013; Micklewright et al., 2015; Anwar et al., 2016; Vaughan et al., 2019) and four revealed a positive relationship (Alipour and Mijani, 2013; Panno et al., 2015; Panno, 2016; Dinç Aydemir and Aren, 2017). It should be noted that two of these studies did not show any relationship between EI and risk behavior through correlation analysis, but the relationship became significant and positive when it was integrated in more complex models involving confounding and mediating variables (Panno et al., 2015; Dinç Aydemir and Aren, 2017). The remaining four articles showed distinct patterns of results as a function of the EI dimension or type of risk studied (Fernández-Abascal and Martín-Díaz, 2015; Lana et al., 2015; Lando-King et al., 2015; Malinauskas et al., 2018). In this regard, the articles of Fernández-Abascal and Martín-Díaz (2015) and Lando-King et al. (2015) reported different results depending on the EI dimension evaluated (they did not compute a global EI score) and although emphasized the existence of a negative relationship between EI and risk behavior, they also found null relationships for some EI dimensions. Likewise, Lana et al. (2015) explored several types of risk behaviors and observed that participants with lower levels of EI had a higher probability of engaging in excessive alcohol consumption and unsafe sex, but no significant effects were found for illicit drug use. Finally, Malinauskas et al. (2018) revealed a positive relationship between EI and traffic risk taking and a negative relationship between EI and substance risk taking. Taken together these results, although there seems to be a tendency toward a negative relationship between EI and risk behavior, the complete review of this literature indicates mixed results. This lack of consistency could be a consequence of the different EI models used and the diversity of risk domains assessed in this field of research. For a better understanding of these findings, we decided to examine the studies by classifying them according to EI model and risk domain.

As shown in Table 1, the studies included in the systematic review have made use of the three different approaches of EI proposed by Joseph and Newman (2010). Focusing on those articles that employed the self-report mixed model, we found that there were three articles showing a negative relationship between EI and risk behavior, two showing a positive relationship, and two showing mainly negative relationships but also null relationships. With regard to the self-report ability model, one article showed a negative relationship, two showed a positive relationship, one showed null relationship and other three showed mixed results (one of these articles also included a mixed model measure). Finally, two articles used the performance-based ability model, one of them showed a negative relationship and the other a positive relationship. Therefore, according to these findings, the relationship between EI and risk behavior do not appear to depend on the EI model employed.

With respect to the risk measures, it is known that risk behavior is a construct that is dependent on the study domain, and it can be classified into domains such as health, social, financial, ethical, or recreational (Weber et al., 2002). By examining the risk domains assessed in each of the articles included in the systematic review and according to the Weber et al. (2002) categorization, we can observe how these articles can be grouped into two main blocks: health-related risk behaviors and financial or gambling-related risk behaviors (see Table 1; we excluded an article that studied risk perception in general). Eight of the articles focused on the study of health-related risk behaviors such as substance abuse, excessive alcohol consumption, sexual risk behavior, risky driving behavior or general health risk behavior. Of these eight articles, three reported exclusively a negative relationship and other three reported mainly negative relationships but also some null relationship. The only cases where EI did not seem to be negatively related to health-related risk behavior was in the field of driving. Two articles worked with risky driving behavior revealing a positive relationship with EI and an absence of relationship (Hayley et al., 2017; Malinauskas et al., 2018). It should be also be noted that the results in the health risk domain did not depend on the EI model (see Table 1). In summary, these results appear to support the existence of a negative relationship between EI and behaviors linked to the health risk domain (with the exception of risky driving). Conversely, the group of six articles employing risk measures related to finances and gambling tasks (two and four studies, respectively), did not revealed a uniform pattern of results. Two articles showed a positive relationship, two showed a negative relationship, and two showed no relationship.

Finally, it is worth noting that none of the articles analyzed the relationship between EI and risk behavior as a function of gender. With respect to age and country of origin of the study, we observed that there does not seem to be a pattern of results associated with these variables (see Table 1).

### Results of the Meta-Analysis

Effect sizes from 12 of the 15 articles included in the systematic review were introduced in the meta-analysis (see Table 1). The three remaining articles were excluded because it was not possible to obtain the required effect sizes from the articles or by request from the corresponding authors. The whole sample of participants for the meta-analysis was *n* = 5,100 (mean percentage of men across studies = 41.98%; mean age across studies = 21.52 years).

**Figure 2:**
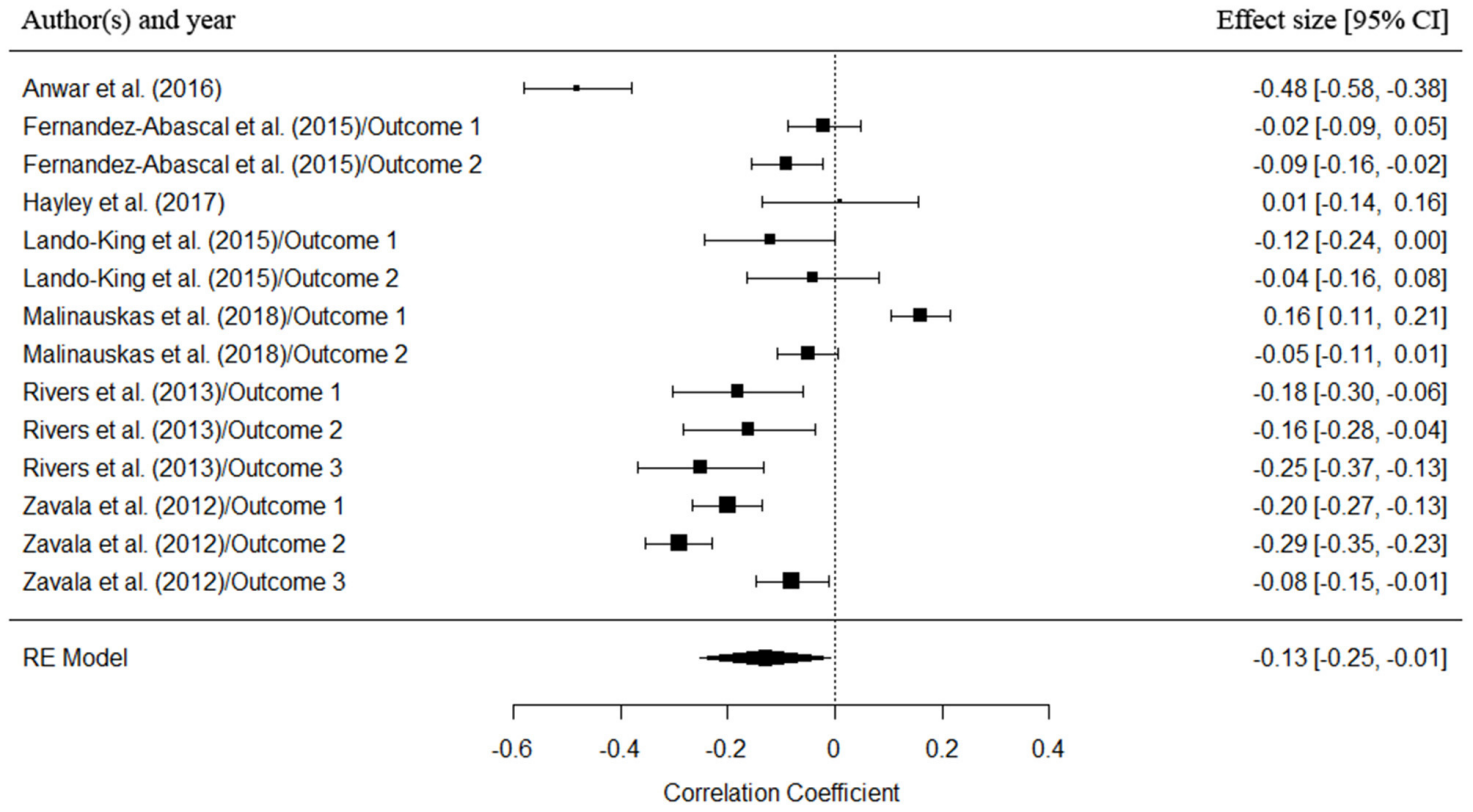
Forest plot displaying the individual and pooled effect sizes (and 95% confidence intervals) of the studies relating EI and health-related risk behaviors included in the meta-analysis. Box sizes represent the weight of each study in the meta-analysis.

The three-level random effects model revealed no significant relationship between EI and risk behavior [estimated effect size = −0.06, *SE* = 0.05, 95% CI [−0.17, −0.04], *p* > 0.05]. Test for heterogeneity suggested the presence of heterogeneity in the sample [*Q*_(18)_ = 248.42, *p* < 0.001]. Since, following the findings of the qualitative synthesis, we have observed that the relationship between these constructs appear to depend on the risk domain studied, we decided to go one step further and include risk domain as a moderator in the meta-analytic model. The two levels of the moderator were health-related risk behaviors and financial/gambling-related risk behaviors. The results for this three-level random/mixed-effects model revealed a significant relationship between EI and health-related risk behaviors [estimated effect size = −0.13, *SE* = 0.06, 95% CI [−0.25, −0.01], *p* = 0.03], but not between EI and financial/gambling domain [estimated effect size = 0.05, *SE* = 0.08, 95% CI [−0.11, 0.21], *p* > 0.05]. The moderating effect of the risk domain factor was marginally significant [*Q*_M(1)_ = 3.29, *p* = 0.06; heterogeneity: *Q*_E(17)_ = 232.10, *p* < 0.001]. In addition, Egger's test did not reveal evidence of possible publication biases (*p* > 0.05) and Rosenberg's Fail-Safe N indicated that 294 additional studies with an effect size of zero would be required to reduce the *p*-value to a non-significant level in the health domain. A forest plot showing the individual and pooled effect sizes (with 95% confidence interval) from the studies relating EI and health-related risk behaviors (i.e., from the significant risk domain) is presented in Figure 2.

## Discussion

The aim of this study was to synthesize existing findings on the relationship between EI and risk behavior in order to advance our understanding of the decision-making process in risk contexts. Importantly, this relationship was studied in terms of the various conceptualizations of EI and risk domains. To this end, we conducted a qualitative and quantitative systematic review of the existing literature.

Fifteen articles studying the relationship between EI and risk behavior were selected for the qualitative analysis after carrying out a systematic search of the literature (April 2020) and applying the inclusion and exclusion criteria described in the Method section. These articles provided a total sample of *n* = 5,461 participants. With respect to the quantitative analysis, 12 out of the 15 articles selected through the systematic review were appropriate and provided the information needed to be included in the meta-analysis (*n* = 5,100). The qualitative analysis revealed that five articles reported a significant negative relationship between global EI and risk behavior, four reported a significant positive relationship, and two reported no relationship. In addition, there were four articles that investigated the relationship between EI and risk through different dimensions of EI (did not report a global EI score) or in more than one type of risk behavior, reporting different results depending on the studied variable. In general, these four articles showed a greater support for the existence of a negative relationship, but null and positive results were also found as a function of the EI dimension and the type of risk. With respect to the results of the quantitative analysis, a three-level random effects meta-analytic model revealed no significant relationship between EI and risk behavior (estimated effect size = −0.06, *p* > 0.05). Preliminary analysis of these findings suggests a rather unclear pattern of results; however, as we describe below, a more in-depth analysis of these studies revealed that these differences depended on certain moderating factors.

When observing the results in more detail, we can appreciate that the articles included in the systematic review used the three EI models proposed by Joseph & Newman (Joseph and Newman, 2010). Negative, positive and null relationships were found for the three EI models, and any trends or patterns did not vary as a function of the model used. Thus, the relationship between EI and risk behavior seem to be independent of the type of EI model employed, at least in these studies. On the other hand, a key factor that does seem to shed light on the discrepancies found in the results is the risk domain. The selected articles primarily focused on two risk domains: risk behaviors associated with health (e.g., alcohol and substance abuse, sexual behavior, and risky driving behavior) and risk behaviors in matters related to finance and gambling. When differentiating between these two domains, we observed a clearer pattern of results for the health-related risk domain. Three of the eight articles studying health-related risk domain showed a significant negative relationship with EI, and other four articles also showed mainly significant negative relationships, although coupled with some positive and null relationships depending on the EI dimension and risk domain studied. The results of the meta-analysis further clarify these findings, revealing that, when risk domain was included as a moderating variable, there was a negative relationship between EI and health-related risk behaviors (estimated effect size = −0.13, *p* = 0.03). The higher the EI levels, the lower the incidence of health risk behaviors. In this regard, EI could act as a protective factor against risk-taking. However, no clear pattern of results was found for the finance/gambling domain, with studies reporting positive, negative, and null relationships (meta-analysis results: estimated effect size = 0.05, *p* > 0.05).

Among the results found for health-related risk behavior it is worth noting the particular case of risky driving behavior. Unlike other risk behaviors associated with health, this type of behavior did not reveal any negative relationship with EI [one article found a positive relationship (Malinauskas et al., 2018) and another a null relationship (Hayley et al., 2017)]. Whilst risky driving behavior is considered a public health risk (WHO, 2018), this behavior has its own particularities that distinguish it from the rest of the risks studied in the health domain. We propose that, although the proneness to taking risks while driving evidently poses a danger to our physical integrity, in this case, the consequences of the behavior may depend more on our skills when compared with other health-related risk behaviors (Megías et al., 2018a,d).

In summary, with the exception of risky driving behavior, our findings support the existence of a negative relationship between EI and risk behavior in the health domain, regardless of the EI model used. Interestingly, in our systematic literature search, previous to apply the exclusion criteria, we found three additional articles that supported these findings. These articles were excluded because they did not use a measurement instrument to specifically evaluate risk behavior. Two of the articles aimed at assessing the level of EI in clinical population groups characterized by problems associated with risk health behaviors, such as illicit drug users and alcohol abusers (Kornreich et al., 2011; Romero-Ayuso et al., 2016). Both studies revealed that the clinical groups had lower levels of EI than the non-clinical groups. In the third article, Goudarzian et al. (2017) showed that EI training can help to reduce the potential use of illicit drugs.

From a theoretical perspective, the relationship between EI and health risk behavior could be understood through the critical role played by emotions in decision making, particularly in risk contexts (Ditto et al., 2006; Gutnik et al., 2006; Rivers et al., 2008; Megías et al., 2011). Many of the risk behaviors associated with health are usually characterized by positive short-term consequences, such as satisfying impulses. Some examples include having unprotected sex for pleasure, drinking more than five or more drinks at a party for fun, riding a motorcycle without wearing a helmet due to considerations of comfort, driving at high speed for adrenaline, or walking through an unsafe area of town in order to take a short cut to our destination. In this type of contexts, the emotion elicited by the short-term rewards can guide our behavior (Cyders and Smith, 2008). This effect is particularly evident if the individual is already in a strong positive or negative emotional state, which increases the influence of the short-term rewards (Cyders and Smith, 2007; Deckman and DeWall, 2011; Smith and Cyders, 2016). Higher emotional abilities, such as a better perception and understanding of our emotions and a greater ability to control them, could act as protective factors against the tendency to be guided by short-term rewards and risk taking in health-related contexts. People with higher levels of EI would be better able to understand and weigh up the health risks in situations with a high emotional burden (Mayer et al., 2001).

The results of our review have also shown that there is no clear evidence supporting the existence of a relationship between EI and risk behavior in the domain of finance and gambling. While we know that people adapt their behavior in risk situations (De Martino et al., 2006; Slovic et al., 2007; Rivers et al., 2008), we also know that the way we adapt our behavior is specific to the risk domain (Weber et al., 2002). Thus, an individual can show a tendency to behave in a risky way in one domain but not in others. There are a wide variety of cognitive and emotional factors that can affect risky decision making and the relative weight of these factors will depend on the contextual situation (Loewenstein et al., 2001; Reyna, 2004; Slovic et al., 2007; Megías et al., 2015). Focusing on the case of financial risk-taking, this type of behavior involves markedly different contextual characteristics in comparison with the previously studied health-related risk behaviors. In the financial context, taking certain risks is unavoidable in the pursuit of economic gains, that is, it is an integral part of the business. In fact, risk taking is considered to be one of the most important aspects of entrepreneurship (Wiklund and Shepherd, 2005). A similar situation could be also occurring in those studies included in the systematic review in which risk behavior was assessed through gambling tasks such as the Iowa gambling task, Columbia card task, and Cambridge gambling task (see Table 1). In these gambling tasks, risk taking, when adopted appropriately, can be necessary for improving performance.^1^ Taken together, these assumptions suggest that the decision to take risks has different consequences in health and financial/gambling contexts, and, therefore, different factors could be involved in the decision-making process. In this regard, the behavioral differences observed in the current review as a function of the context where the risk is performed are in accord with the domain specificity of risk behavior (Weber et al., 2002).

The results of the present study are not exempt from some limitations. The articles included in the systematic review only focused on the risk domains of health and finance/gambling, and in the latter case only six articles were found. With the objective of gaining a more complete understanding of the influence of EI on risk decision making, further research should focus on other risk scenarios such as those in social, recreational, and ethical contexts (Blais and Weber, 2006). In order to increase the generalizability of the findings, it will also be necessary to address possible gender and age differences. Moreover, future studies should employ experimental designs to examine causality and, thus, establish the possible protective role of EI in health risk behavior. Finally, we must also consider some intrinsic limitations of the measurement instruments used in the literature reviewed. A number of different EI and risk measures were included, each of them with very different characteristics (e.g., overall scores vs. dimensional scores, self-report vs. performance-based measures, different EI models and risk domains, etc.), which hinders extrapolation of the results. For example, as previously mentioned, risk situations are highly emotionally charged, which could bias self-report measures, since the responses of individuals in hypothetical situations (without exposure to the emotional burden) can be somewhat different to the responses elicited in context closer to real situations. Further, it is recommended that future research studies focus on performance-based ability measures of EI, such as the MSCEIT (Mayer et al., 2002). Most of the studies included in this review (13 of the 15) used self-report EI measures. Although these instruments present a greater ease and speed of administration, previous research has shown that the performance-based ability model, in comparison with self-report ability and mixed models, has better divergent validity and greater predictive ability for performance in emotionally charged cognitive tasks and general behavior (Gutiérrez-Cobo et al., 2016; Mayer et al., 2016; Megías et al., 2017).

In conclusion, the results of this systematic review and meta-analysis contribute toward achieving an in-depth understanding of the relationship between EI and engagement in risk behavior in various settings. The findings obtained from our search of the literature support the notion that risk is a domain-specific construct (Weber et al., 2002). In particular, the relationship between EI and risk behavior differed according to the risk domain studied; a negative relationship was found when studying the health domain, whilst this relationship was unclear in the financial and gambling domain. The results associated with the health domain are consistent with existing literature about the positive impact of emotional abilities on the optimal health and wellbeing of individuals (Schutte et al., 2007; Laborde et al., 2014; Fernández-Berrocal and Extremera, 2016). In situations where our health can be put at risk, EI abilities could play an important role in protecting against the tendency to engage in risk behaviors. Given the considerable impact of risk-taking on public health, a better understanding of the mechanisms underlying the relationship between EI and risk behavior could help to inform the development of intervention programmes aimed at preventing and reducing the negative effects of these behaviors on our society.

## Data Availability Statement

The original contributions presented in the study are included in the article/supplementary material, further inquiries can be directed to the corresponding author.

## Author Contributions

AM-R, MTS-L, and PF-B: design study. MTS-L and AM-R: database search, comprehensive reading of selected articles, and writing. All authors: selection of final articles and review. AM-R: statistical analysis. All authors contributed to the article and approved the submitted version.

## Funding

This work was funded by the Regional Ministry of Economy and Knowledge, Junta de Andalucía (project: EMERGIA20_00056 and UMA18-FEDERJA-137) to AM-R, by the Spanish Ministry of Economy, Industry and Competitiveness (project: PSI2017-84170-R) to PF-B, and by the Spanish Ministry of Education and Vocational Training (to FPU15/05179 RG-L and FPU18/00610 to MTS-L).

## Conflict of Interest

The authors declare that the research was conducted in the absence of any commercial or financial relationships that could be construed as a potential conflict of interest.

## Publisher's Note

All claims expressed in this article are solely those of the authors and do not necessarily represent those of their affiliated organizations, or those of the publisher, the editors and the reviewers. Any product that may be evaluated in this article, or claim that may be made by its manufacturer, is not guaranteed or endorsed by the publisher.

## Footnotes

^1^At this point, it is important to differentiate between the nature of the gambling tasks that have been reviewed (e.g., Iowa gambling task) and other types of behaviors associated with gambling disorder, which are more strongly linked to health-related risk behaviors and have not been addressed in the articles included in this systematic review (Navas et al., 2017).
